# Evidence for Layered Quantized Transport in Dirac Semimetal ZrTe_5_

**DOI:** 10.1038/s41598-018-23011-3

**Published:** 2018-03-23

**Authors:** Wei Wang, Xiaoqian Zhang, Huanfeng Xu, Yafei Zhao, Wenqin Zou, Liang He, Yongbing Xu

**Affiliations:** 1 National Laboratory of Solid State Microstructures, School of Electronic Science and Engineering and Collaborative Innovation Center of Advanced Microstructures, Nanjing University, Nanjing, 210093 China; 2 Department of Electronics, York-Nanjing Joint Centre (YNJC) for spintronics and nano engineering, the University of York, York, YO10 3DD United Kingdom

## Abstract

ZrTe_5_ is an important semiconductor thermoelectric material and a candidate topological insulator. Here we report the observation of Shubnikov-de Hass (SdH) oscillations accompanied by quantized Hall resistance in bulk ZrTe_5_ crystal, with a mobility of 41,000 cm^2^V^−1^s^−1^. We have found that the quantum oscillations does not originate from the surface states, but from the bulk states. Each single layer ZrTe_5_ acted like an independent 2D electron system in the quantum Hall regime having the same carrier density and mobilities, while the bulk of the sample exhibits a multilayered quantum Hall effect.

## Introduction

The layered ZrTe_5_ crystal, with large thermopower^1–5^, has been studied by scientists for many years. Recently, it has attracted more attention after been predicted as a 2D topological insulator(TI) with a bulk direct band gap of 0.4 eV^6^. But experimentally, the topological nature of ZrTe_5_ is still under debate. Some studies reported ZrTe_5_ as a weak TI^7,8^. While, several other experimental studies have suggested that ZrTe_5_ might be a Dirac semimetal^9,10^. The discrepancy may come from the fact that ZrTe_5_ is in a topological critical state placed between a weak-TI and a strong-TI which is sensitive to the lattice constant. The lattice constant is influenced by the growth conditions and the measurement environments.

In this work, we have studied the quantum oscillations of bulk ZrTe_5_ grown by the chemical vapor transport method (CVT) using iodine as the transport agent. Quantum oscillations from the bulk states have been observed having a high mobility of 41000 cm^2^V^−1^s^−1^. The Fermi surface was shown to be two-dimensional with a Berry phase of π in the infinite field limit, which indicated that ZrTe_5_ is a topologically non-trivial material. More importantly, we have found quantized Hall resistance with a filling number of *ν* = *n* + 1/2, and quantized step size of ~e^2^/h per monolayer. Both SdH oscillations and quantized Hall resistance suggest that ZrTe_5_ is a 2D non-trivial material with weak inter-layer interactions.

## Results

### Electronic structure of ZrTe_5_

**Figure 1:**
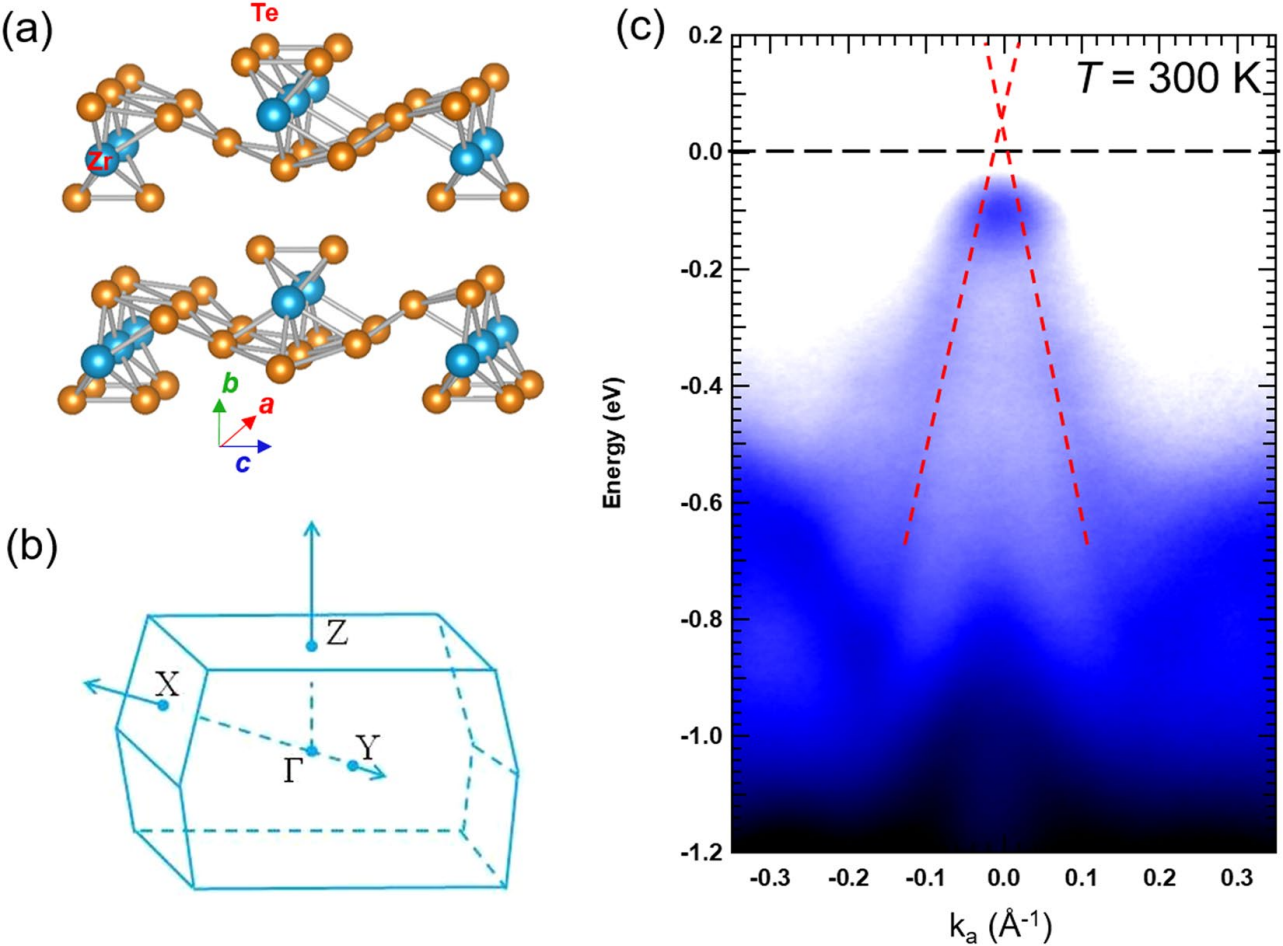
The electron structure of ZrTe_5_. (**a**) Crystal structure of ZrTe_5_. (**b**) The Brillouin zone of ZrTe_5_. (**c**) The ARPES image of the ZrTe_5_ at 300 K. The linear E-K relationship is indicated by red dashed lines.

As shown in Fig. 1a, the structure of the transition-metal Pentatelluride ZrTe_5_ exhibits a quasi-two-dimensional structure (space group is *Cmcm*). Within the *a-c* plane, zigzag chains of Te atoms along the *a*-axis are linked to trigonal prismatic chains of ZrTe_3_ running along the *c*-axis. The 2D planes bond weakly via van der Waals forces along the *b*-axis, forming the 3D bulk crystal.

To verify the band structure of ZrTe_5_ crystal, we performed angle-resolved photoemission spectroscopy (ARPES) measurements, using a photon energy of 21.2 eV (He I α resonance line). Figure 1c shows the electron E-K diagram around the center of the Brillouin zone (Fig. 1b). Near the Γ point, we measured a linear E-K dispersion (as indicated by the red dashed lines in Fig. 1c), suggesting the presence of Dirac fermions. At the Γ point, the Fermi level is very close to the top of the valence band at 300 K, which implies a hole dominated electronic structure, consistent with previous transport measurement work^10^.

### Temperature-dependence of the longitudinal resistivity of ZrTe_5_ crystal

**Figure 2:**
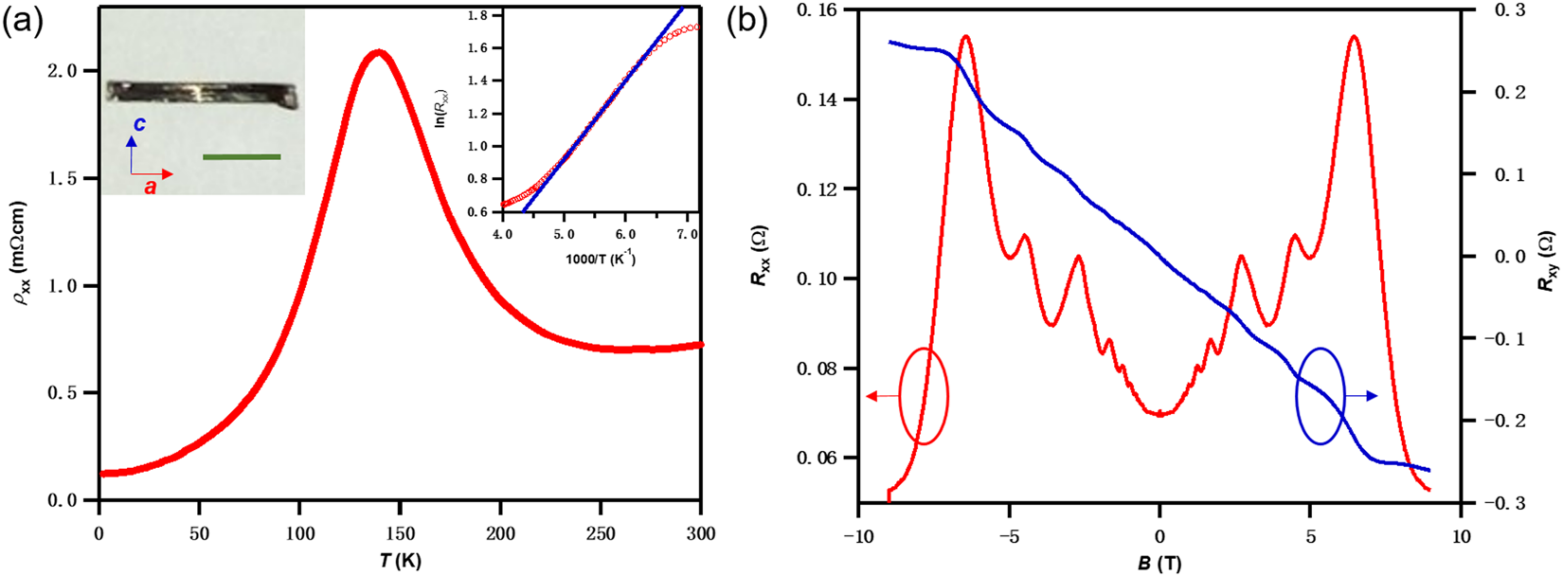
The R-T relationship and the magnetoresistance *R*_xx_ and Hall resistance *R*_xy_ at 2 K. (**a**) The temperature dependent resistivity of the ZrTe_5_ sample. The current is parallel to the crystalline needle axis (I//a). Left Inset: The optic image of an as-grown bulk crystal, the scale bar is 1 mm. Right Inset: The Arrhenius plot, which yields an activation energy of 41 meV in the temperature range of 160–200 K. (**b**) The magnetoresistance *R*_xx_ and Hall resistance *R*_xy_ at 2 K. Quantum oscillations can be observed in *R*_*xx*_, as well as plateaus in *R*_*xy*_.

Figure 2a shows the longitudinal resistivity as a function of temperature for the ZrTe_5_ sample. At room temperature, the resistivity is ~0.7 mΩ·cm, suggesting a poor semimetal. As the temperature decreases from 300 K, the resistivity increases exponentially like that of a semiconductor. The activation energy can be estimated from the Arrhenius plot of ln(*ρ*_xx_) vs 1/*T* for high temperatures, as shown in Fig. 2a (right inset). We measure an activation energy of 41 meV, which is close to the Fermi level position in the work by Shaochun Li *et al*.’s result^7^. At *T = *140 K, the resistivity reaches a maximum. This is also accompanied with the changing of carriers from hole-dominated to electron-dominated as the temperature decreases.

This resistivity peak, also known as the metal-insulator transition, has long been observed in various experimental reports, and the transition temperature ranges from 60 K to 145 K^9^^–^^11^, depending on the detailed growth conditions. The origin of this transition has puzzled scientists for many years and the mechanism is still under debate.

### Quantum oscillations in ZrTe_5_ crystals

Figure 2b shows the longitudinal resistance *R*_xx_ and the Hall resistance *R*_xy_ as the functions of the perpendicular magnetic field (*B*, applied along the *b*-axis) at *T* = 2 K. The sample is *n*-type, with a carrier density *n*_3D_^Hall^ = 1.8 × 10^18^ cm^−3^ as measured from the slope at low field. We also observe an *n-p* transition temperature around *T* = 140 K, which is in agreement with previous work^12^. Pronounced oscillations in *R*_xx_ can also be observed associated with quantized plateaus in *R*_xy_. These are the quantum oscillations from the quantized Landau levels at high magnetic field. Such plateaus are very similar to the quantum Hall effect (QHE) observed in low carrier density and high mobility systems. In the ZrTe_5_ crystal structure, the trigonal prismatic chains of ZrTe_3_ run along the *a*-axis, forming a 2D sheet of ZrTe_5_ in *a-c* plane (Fig. 1a). Because the interaction between the ZrTe_5_ layers is weak^6^, each layer ZrTe_5_ provided an independent 2D conduction channel, as discussed later.

**Figure 3:**
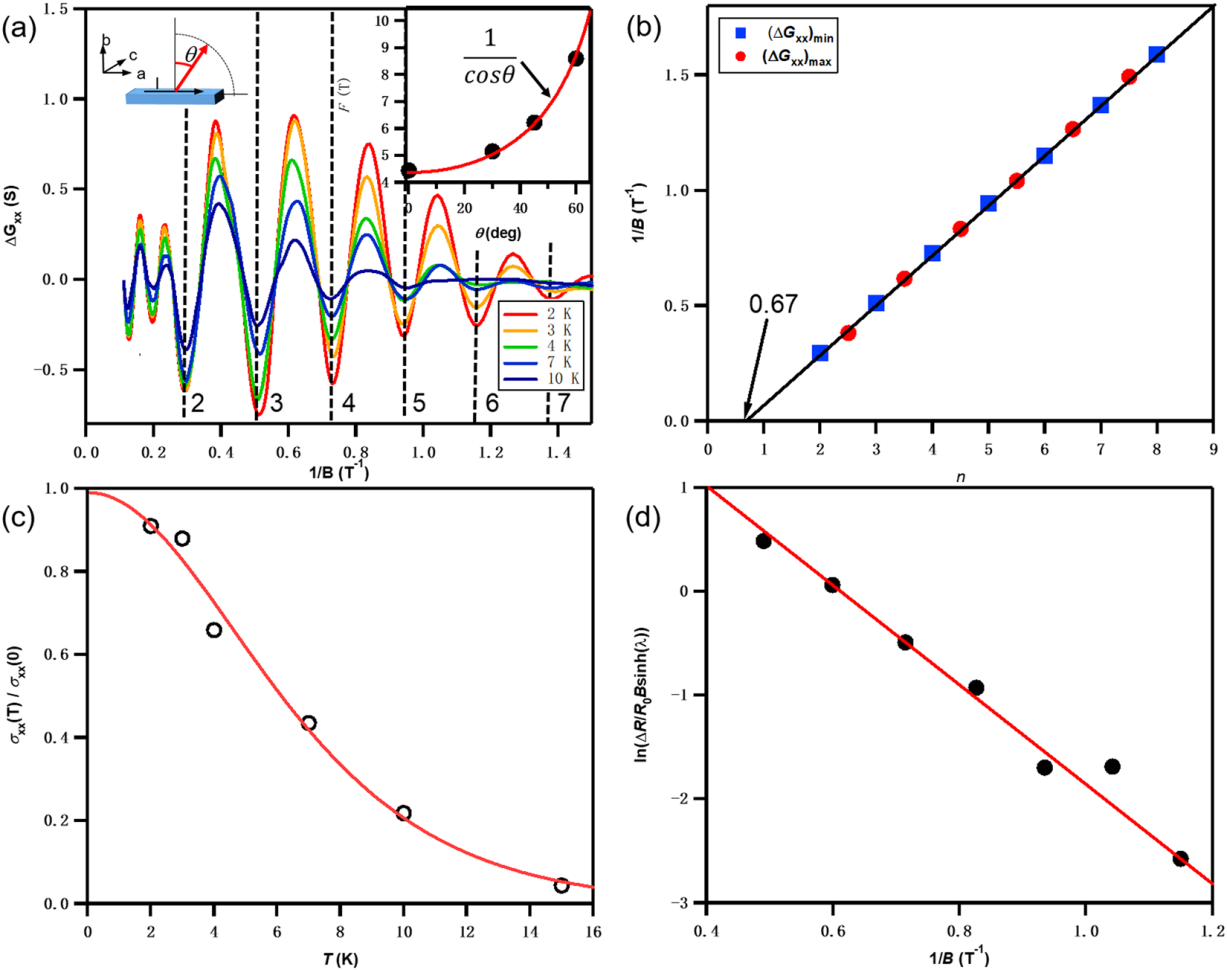
Quantum oscillation of ZrTe_5_. (**a**) Shubnikov-de Hass oscillatory components at various temperatures. The oscillation frequency is ~4.6 Tesla. Right inset: The angle dependence of the oscillation frequency *f*. (**b**) Landau-level fan diagram. The linear fitting gives a nonzero intercept of 0.67, suggesting the Berry phase is close to π. (**c)** Temperature dependence of the normalized amplitude Δ*σ*_*xx*_(*T*)/Δ*σ*_xx_(0). The solid red line is the best fit to *λ*(*T*)/sinh(*λ*(*T*)). The magnetic field of 3.4 T was used to extract the cyclotron mass: *m*_cyc_ = 0.08 m_e_. **(d)** The Dingle plot of ln[(Δ*R*/*R*_*0*_*)B*sinh(*λ*)] versus 1/*B* at 2 K.

Here, we calculate the magnetic conductance *G*_xx_ = *R*_□_/(*R*_xy_^2^ + *R*_□_^2^), where *R*_□_ is the sheet resistance. After subtracting the non-oscillating background, the oscillatory parts of *G*_xx_ (Δ*G*_xx_) display periodic peaks (maxima) and valleys (minima) as a function of 1/*B*, where *B* is the magnetic field intensity. Figure 3a shows the temperature dependence of the SdH oscillations in Δ*G*_xx_. The amplitudes of the oscillations decrease with increasing temperature, up to 10 K. The FFT analysis of the oscillations shows a single frequency *f* = 4.6 Tesla. The Onsager’s formula gives *f* in terms of the cross section area of the Fermi surface (*A*_*F*_) in the momentum space:

1 $$f=\frac{h}{4{\pi }^{2}e}{A}_{F}$$

where *h* is the Planck constant, and *e* is the electron charge. For 2D carrier density: *n*_*2D*_ = *k*_*F*_^2^*/4π*. By substituting the frequency *f* of 4.6 Tesla, the Fermi vector *k*_F_ can be determined as 0.012 Å^−1^. The 2D carrier density *n*_*2D*_ is 1.1 × 10^11^ cm^−2^.

Figure 3a (right inset) shows the oscillation frequencies at different out-of-plane magnetic field direction *θ*. The 1/cos *θ* dependence, suggests the 2D nature of the Fermi surface.

Figure 3b shows the Landau level fan diagram. The maxima and the minima of the *G*_*xx*_ in Fig. 3a, are represented by the blue circles and red squares, respectively. The Lifshitz-Onsager quantization rule shows that ${S}_{F}\frac{\hslash }{eB}=2\pi (n+\frac{1}{2}+\beta +\delta )$, where 2π*β* is the Berry phase and 2π*δ* is the additional phase shift. For linear energy distribution Dirac fermions, the Berry phase should be π (*β* = 1/2). *δ* is determined by the dimensionality of the Fermi surface and the value changes from 0 for surface states (2D) to ±1/8 for bulk states (3D)^13^. The linear fitting of our data yields a finite intercept of 0.67 which is very close to the value of 1/2 + 1/8 = 0.625. This result suggests a non-trivial bulk channel, that is different from strong 3D topological insulator surface states^14,15^.

According to the Lifshitz-Kosevich (LK) theory^16^, we can calculate the effective cyclotron mass *m*_*cyc*_ = 0.08 m_e_ from the temperature dependence of the SdH oscillation amplitude. The Fermi velocity is calculated as ${\nu }_{F}=\hslash {k}_{F}/{m}_{cyc}=1.7\times {10}^{5}\,{{\rm{ms}}}^{-1}$, and the Fermi level *E*_*F*_ = *m*_*cyc*_*ν*_*F*_^2^ = 13 meV above the Dirac point, considering that electrons are the majority carrier type. Figure 3d shows the Dingle plot of ln[Δ*R*/*R*_0_*B*sinh(*λ*)] versus 1/*B*. The slope is used to calculate the quantum scattering time *τ* = 1.89 × 10^−12^ s. Thus the mean-free path of electrons is *l* = *ν*_F_*τ* = 3.2 × 10^−7^ m, which in turn gives an estimate of the carrier mobility *μ*_SdH_ = *eτ/m*_cyc_ = 41000 cm^2^V^−1^s^−1^.

### 2D-like bulk quantum Hall effect

**Figure 4:**
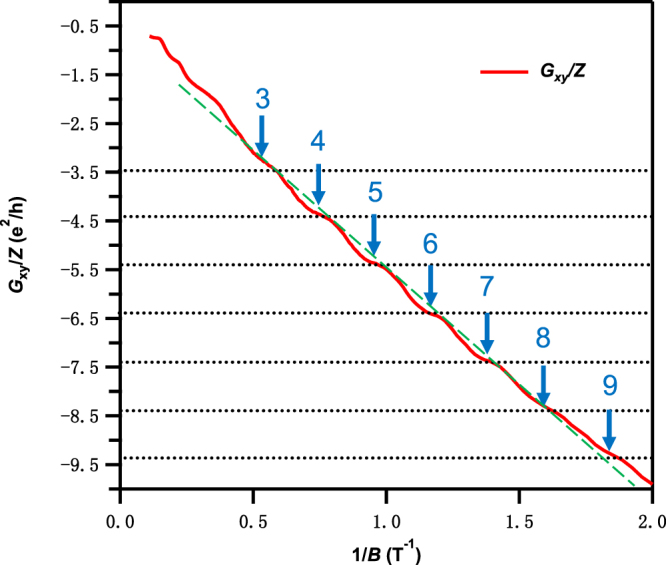
The bulk quantum Hall effect of ZrTe_5_. *G*_xy_ divided by the number (*Z*) of layers plotted as a function of 1/*B*, displaying quantized plateaus separated by ~1 e^2^/h between adjacent LLs.

In Fig. 4, we plot *G*_*xy*_ divided by the number of layers (*Z*) in our sample as a function of 1/*B*, *Z* = 1.4 × 10^5^ is calculated from the measured bulk thickness divided by the thickness of a ZrTe_5_ monolayer. Surprisingly, the plateaus display a linear relationship with 1/*B*, as indicated by the green dashed line in Fig. 4. Also, the step size between the plateaus is approximately 1 e^2^/h. This suggests that the plateaus are indeed Landau levels developed under the influence of the magnetic field.

More importantly, the Landau level filling number can be indexed as *ν* = *n* + 1/2, instead of *n*. This half-integer quantization essentially stems from the existence of the zeroth Landau level for Dirac fermions, similar to the reported bulk QHE in 3D topological insulators Bi_2_Se_3_^17^. This provides further evidence that ZrTe_5_ is a topologically non-trivial material.

## Discussion

According to our measurements, both the intercept of the Landau level fan diagram and the electron structure prove that the ZrTe_5_ crystal is a topologically non-trivial material. But unlike other 3D Dirac semimetals such as Cd_2_As_3_, we observe that the quantum Hall plateaus in *G*_xy_/*Z* and the step size is approximately 1 e^2^/h. Similar phenomena have been observed in bulk QHE systems, like: GaAs/AlGaAs superlattice^18^, Mo_4_O_11_^19^ and Bechgarrd salts^20–22^. We also estimate the total carrier density as *n*_total_ = *n*_2D_/*d* = 1.1 × 10^11^ cm^−2^ × 1.38 × 10^17^ cm^−1^ = 1.5 × 10^18^ cm^−3^ (*d* is the thickness of monolayer ZrTe_5_). This number is very close to the calculated Hall density of 1.8 × 10^18^ cm^−3^. According to Hongming Weng *et al*.’s previous work^6^, the ZrTe_5_ crystal has much lower interlayer binding energy than Bi_2_Se_3_ and Bi(111) bilayers. That not only means the ZrTe_5_ monolayers are easier to be exfoliated by scotch tape, but it also means that ZrTe_5_ behaves as a series of stacked parallel 2D conduction channels. Other experiments have also shown this. For example, in Yanwen Liu *et al*.’s work, they extracted the disk-like Fermi surface of ZrTe_5_ from the angle dependent SdH oscillations, which indicated that ZrTe_5_ has a 2D-like band structure. In this case, we believe the QHE of our ZrTe_5_ is due to transport through many parallel 2D conducting channels formed by the ZrTe_5_ monolayers. Such bulk QHE was also observed in the heavily doped *n*-type Bi_2_Se_3_^17^.

Thus, our analysis suggests that the inter-layer interaction of the bulk ZrTe_5_ is relatively weak, and that ZrTe_5_ is more likely to be a Dirac Semimetal, rather than a weak-TI.

## Methods

### Sample preparation

Single crystals of ZrTe_5_ was prepared from 99.99% Zr and 99.999% Te purchased from Alfa Aesar. Single crystals were obtained by means of chemical transport reactions, using iodine as the transport agent.

### Electrical measurements

The longitudinal and transvers resistance *R*_*xx*_ and *R*_*xy*_ were measured by a standard six-point Hall bar geometry in a Quantum Design physical properties measurement system (PPMS-9T). The electrical characteristics were measured using resistivity option with a current of 10 μA.

### ARPES measurement

Our ARPES data were taken with a PHOIBOS 150 Hemispherical Energy Analyzer at room temperature. A He I α (21.2 eV) resonance emission line, from a high flux UVS300 He lamp was used to excite the photoelectrons from the sample surface. The UV radiation angle of incidence was 45° which relative to the sample normal and the spot size was 0.5 mm × 1 mm. All of the photoelectron measurements were performed with an angular resolution better than 0.2° in the wide-angle mode (15°) of the analyzer while the analyzer energy resolution was 30 meV.
